# Mitochondrial DNA Signature for Range-Wide Populations of *Bicyclus anynana* Suggests a Rapid Expansion from Recent Refugia

**DOI:** 10.1371/journal.pone.0021385

**Published:** 2011-06-22

**Authors:** Maaike A. de Jong, Niklas Wahlberg, Marleen van Eijk, Paul M. Brakefield, Bas J. Zwaan

**Affiliations:** 1 Institute of Biology, Leiden University, Leiden, the Netherlands; 2 Department of Biosciences, University of Helsinki, Helsinki, Finland; 3 Department of Biology, University of Turku, Turku, Finland; 4 Department of Zoology, University of Cambridge, Cambridge, United Kingdom; 5 Laboratory of Genetics, Wageningen University, Wageningen, the Netherlands; Rutgers University, United States of America

## Abstract

This study investigates the genetic diversity, population structure and demographic history of the afrotropical butterfly *Bicyclus anynana* using mitochondrial DNA (mtDNA). Samples from six wild populations covering most of the species range from Uganda to South Africa were compared for the cytochrome c oxidase subunit gene (COI). Molecular diversity indices show overall high mtDNA diversity for the populations, but low nucleotide divergence between haplotypes. Our results indicate relatively little geographic population structure among the southern populations, especially given the extensive distributional range and an expectation of limited gene flow between populations. We implemented neutrality tests to assess signatures of recent historical demographic events. Tajima's *D* test and Fu's *F_S_* test both suggested recent population growth for the populations. The results were only significant for the southernmost populations when applying Tajima's *D*, but Fu's *F_S_* indicated significant deviations from neutrality for all populations except the one closest to the equator. Based on our own findings and those from pollen and vegetation studies, we hypothesize that the species range of *B. anynana* was reduced to equatorial refugia during the last glacial period, and that the species expanded southwards during the past 10.000 years. These results provide crucial background information for studies of phenotypic and molecular adaptation in wild populations of *B. anynana*.

## Introduction

The degree of isolation and the demographic history of populations are key factors influencing the potential of populations to adapt to divergent environmental conditions. Understanding the spatial genetic structure of populations is crucial for making inferences about adaptive geographic variation in species, and their adaptive potential to respond to future changes in the environment. Species-specific life history characteristics can influence geographic population structure, for example, an increased dispersal capacity leads to higher level of gene flow, which can slow down or limit geographic differentiation [1]. Historical biogeographical changes can have an effect on the genetic variation within and among populations. For instance, past habitat reduction can cause a demographic bottleneck and a decrease in genetic variation, thereby decreasing the ability of a population to respond to selection. Variation in the genome, on the population and the geographic level, bears the footprints of past demographic events as well as ongoing and current population genetic processes such as gene flow. Studying the molecular phylogeny and population genetics of a species can reveal evidence of past biogeographic events and suggest life history traits that contribute to shaping the distribution of genetic variation among populations [2].

The afrotropical butterfly *Bicyclus anynana* (Nymphalidea) is increasingly used as a model species in evolutionary genetics and life-history studies [3]–[5], but so far intra-specific phylogeographic information based on molecular data is lacking. The species inhabits the Eastern part of sub-Saharan Africa, where its range stretches from the equator to the subtropics, spanning an area of more than 3000 kilometers. This region is largely dominated by savannah vegetation and characterized by a strong seasonality in rainfall, although the intensity and frequency of the alternating wet and dry seasons vary according to latitude. The preferred habitat of *B. anynana* is the edges of the dry forests that occur along the rivers, lakes and the coast of the savannah area, where the adults feed on fallen forest fruit, while the larvae develop on grasses [6]. The species copes with the seasonal nature of its habitat by expressing alternate adult phenotypes as an adaptation to the contrasting seasonal environments [7], [8]. This phenotypic plasticity enables survival of the harsh dry seasons without the need to migrate or diapause. The adult butterflies are weak flyers, generally resting in the shade during the warmest part of the day, and flying mainly during the morning and late afternoon. Although *B. anynana* is a relatively common species, its habitat is naturally very fragmented, and is decreasing rapidly in many regions due to man-induced habitat loss and degradation.

In this study, we use mitochondrial DNA (mtDNA) sequence data to examine the phylogeography of *B. anynana*. mtDNA is widely used as a tool in phylogeographic studies, due to its low or absent recombination, uniparental inheritance, conserved structure and relatively high evolutionary rate [2], [9], [10]. The analysis of intraspecific mtDNA variation can reveal information about the interconnectivity of populations and past demographic events such as population expansions [2]. The cytochrome oxidase I (COI) gene is one of the most frequently employed mtDNA genes to investigate phylogeographic patterns and histories at the inter- and intraspecies level, and has been extensively used for evolutionary studies in insects [11]. We investigate the range-wide phylogeography of *B. anynana* using six populations distributed throughout the species' range, and including a population of a different subspecies and an island population. We sequenced 1500 bp of the COI gene for 25 individuals per population, and focused the analysis on: 1) the sequence diversity and variability, 2) population subdivision, and 3) the demographic history of the populations. The main aim of this study is to provide information on the isolation of the populations and their phylogeographic history, which will be especially interesting in the light of ongoing and future studies of adaptive phenotypic and genetic geographic variation in the species.

## Materials and Methods

### Sample collection

**Figure 1:**
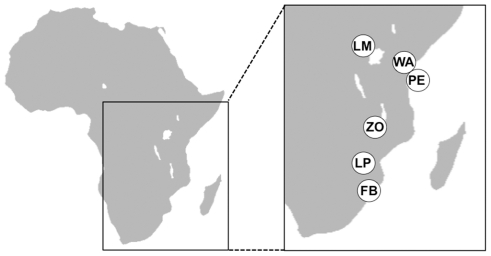
Overview of the locations of the studied populations on the African continent.

We took a sample of 25 adults of *Bicyclus anynana* from each of six populations. The samples were collected in 2005 and 2006 from the following locations (followed by their abbreviations and geographic coordinates): Lake Mburo in Uganda (LM, 0°38′S 30°57′E); Watamu in Kenya (WA, 3°21′S 40°1′E); Ngezi forest on Pemba Island, Tanzania (PE, 4°55′S 39°42′E); Zomba in Malawi (ZO, 15°22′S 35°19′E); Mpaphuli Cycad Reserve in Limpopo, South Africa (LP, 22°47′S 30°37′E); and False Bay Park of the Greater St Lucia Wetland Area, KwaZulu Natal, South Africa (FB, 27°58′S 32°21′E). A schematic map of Africa with the locations of the populations is given in Figure 1. Butterflies were frozen alive at −80°C and stored at the same temperature until they were further used for DNA extraction.

### DNA extraction, PCR and sequencing

Genomic DNA was extracted from individual thoraces and legs using Qiagen's DNeasy tissue kit, following the manufacturer's instructions. The COI gene was amplified in two parts with a total length of ∼1500 bp, using two universal primer pairs as described in [12]. The polymerase chain reaction (PCR) was conducted in a total volume of 20 µl, containing 1.0 µl of DNA template, 2.0 µl 10× buffer, 2.0 µl MgCl2 (25 mM), 1.0 µl primer 1 (10 mM), 1.0 µl primer 2 (10 mM), 0.4 µl dNTPs (10 mM), 0.1 µl taq polymerase (5 U/µl) and 12.5 µl Milli-Q water. PCR conditions consisted of an initial cycle at 95°C for 5 min, 40 cycles of 94°C for 30 sec, 50°C for 30 sec, 72°C for 90, and a final extension at 72°C for 10 min. PCR products that yielded a clear band on agarose gel by electrophoresis were purified and sequenced using capillary electrophoresis sequencing by Macrogen Europe.

### Data analysis

#### Variation within and among populations

**Figure 2:**
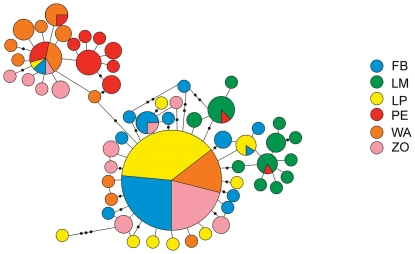
Haplotype network. Circle size is relative to number of haplotype copies present in dataset. A branch represents a single nucleotide change, black dots on branches represent inferred missing haplotypes (single nucleotide changes).

Individual sequences were aligned manually using the program BioEdit 7.0.9.0 [13]. All statistical parameters and tests were calculated using the program Arlequin 3.5 [14]. Genetic diversity within populations was estimated by computing haplotype diversity (*H*) and nucleotide diversity (π) [15]. Haplotype diversity (also known as gene diversity) represents the probability that two randomly sampled alleles are different, while nucleotide diversity Is defined as the average number of nucleotide differences per site in pairwise comparisons among DNA sequences [15]. Relationships between haplotypes were estimated using the minimum spanning network method (also called molecular-variance parsimony technique). The haplotype network was computed under haplotype pairwise differences, giving the number of mutation steps between haplotypes. The network was subsequently drawn by hand (Figure 2). Partitioning of genetic variation within and among populations was calculated using analysis of molecular variance (AMOVA) [16], by computation of conventional *F*-statistics from haplotypes with 1000 permutations.

#### Neutrality and demographic history

Statistical tests originally developed to assess the selective neutrality of mutations have been implemented to test for demographic expansion in recent years [17]. These tests are designed to distinguish between neutrally evolving sequences under mutation-drift equilibrium, and sequences evolving under non-neutral processes including directional or balancing selection, and demographic expansion or contraction. In order to test for past population expansion, we used two statistical tests commonly used to analyze demographic events. Tajima's *D* uses the frequency of segregating nucleotide sites, while Fu's *F_S_* [18] uses the distribution of alleles or haplotypes. Both tests are based on the principle that a sudden population expansion that is associated with a non-neutral process will show a shift in the allele frequency spectrum compared to a neutral Wright-Fisher model consistent with population expansion under neutral evolution. The analyses were implemented in the program Arlequin 3.5 [14], p-values were generated using 1000 simulations under a model of selective neutrality.

## Results

### Diversity indices

We analysed a combined length of 1480 bp of the COI gene for a total of 150 individuals from six wild *B. anynana* populations. Over the whole data set, we identified 54 polymorphic sites leading to the definition of 59 haplotypes. One haplotype was found in four populations (FB, LP, ZO and WA) and occurred frequently (21%) over the total data set. In addition to this common haplotype, six other haplotypes were shared by at least two populations. The shared haplotypes represented 47% of the total number of individuals. Finally, 52 were private (unique to a single population), most of them being singleton haplotypes. The number of haplotypes was comparable among populations, varying between 10 for LP to 13 for FB (Table 1). The genetic diversity was large in every population, ranging from *H* = 0.75 to *H* = 0.92 (Table 1), with a mean gene diversity per population of *H* = 0.866. In contrast, nucleotide diversity was relatively low for each population, ranging between π = 0.0015 for LP and π = 0.0023 for LM (Table 1). Although overall diversity was similar among populations, gene diversity and nucleotide diversity were both lowest for LP, followed by the island population PE. Gene diversity was highest for FB, while LM showed the highest nucleotide diversity (Table 1).

**Table 1:** Sample size (n), number of haplotypes (k), number of polymorphic sites (PS), haplotype diversity (*H*) ± SD and nucleotide diversity (π) ± SD per population.

Population	n	k	PS	*H*	π
False Bay	25	13	18	0.897±0.043	0.0018±0.0011
Limpopo	25	10	19	0.747±0.082	0.0015±0.0009
Zomba	25	12	14	0.883±0.051	0.0019±0.0012
Pemba	25	11	15	0.860±0.050	0.0017±0.0010
Watamu	25	12	12	0.917±0.032	0.0018±0.0011
Lake Mburo	25	12	14	0.893±0.038	0.0023±0.0014

### Geographical structure

Results from the AMOVA showed that the overall genetic variation within populations (65.16%) was much larger than the variation among populations (34.84%). Pairwise *F_ST_* values between populations were all significant except for the comparison between the two southernmost populations FB and LP, which had a very low *F_ST_* value of 0.008 (Table 2). The island population PE and the *centralis* subspecies population LM showed most differentiation in pairwise comparisons with the other populations. The highest pairwise *F_ST_* value resulted from the comparison between these two populations (*F_ST_* = 0.576). PE showed the least differentiation compared to WA, which is geographically the nearest population on the mainland. For the mainland populations FB, LP, ZO and WA, pairwise *F_ST_* values generally increased with geographic distance, with the highest *F_ST_* values between the southern populations FB and LP compared with WA (Table 2).

**Table 2:** AMOVA haplotype *F_ST_* results for pairwise population comparisons (lower diagonal) and associated significance indications (upper diagonal).

	FB	LP	ZO	PE	WA	LM
**FB**	-	NS	**	***	***	***
**LP**	0.008	-	**	***	***	***
**ZO**	0.066	0.080	-	***	**	***
**PE**	0.494	0.532	0.376	-	***	***
**WA**	0.326	0.369	0.168	0.212	-	***
**LM**	0.265	0.260	0.348	0.576	0.487	-

FB: False Bay; LM: Lake Mburo; LP: Limpopo; PE: Pemba; WA: Watamu; ZO: Zomba; NS: not significant; ** p<0.01; *** p<0.001.

The haplotype network (Figure 2) clearly reflects the results of the diversity indices and AMOVA presented above. It shows the common haplotype which was present in four of the populations, with many single haplotypes around it. The star-like shape of this part of the network indicates little geographical structure, in particular for the mainland populations FB, LP and ZO. For these populations the network shows very low levels of sequence divergence and a high frequency of unique mutations, which can be a signature of rapid population expansion. The haplotypes of the WA, and especially the PE and LM, samples form more distinct groups and hence show more geographic structure, although sequence divergence is generally low.

### Demographic analysis

The results of Tajima's *D* test and Fu's *F_S_* test are presented in Table 3, including associated simulated p-values. Tajima's *D* values were negative for all populations, indicating an excess of rare nucleotide site variants compared to the expectation under a neutral model of evolution. Only for the two southernmost populations, FB and LP, were these deviations from neutrality significant. The results of Fu's *F_S_* test, which is based on the distribution of haplotypes, also show negative values for all populations, indicating an excess of rare haplotypes over what would be expected under neutrality. Following this test, the hypothesis of neutral evolution was significantly rejected for all populations except for the *centralis* subspecies population LM.

**Table 3:** Results of Tajima's *D* and Fu's *F_S_* neutrality tests including associated p-values.

Population	Tajima's *D*	p	Fu's *F_S_*	p
False Bay	−1.66	0.037	−6.22	0.002
Limpopo	−2.10	0.008	−3.50	0.026
Zomba	−0.87	0.176	−4.41	0.017
Pemba	−1.40	0.058	−4.04	0.014
Watamu	−0.68	0.291	−5.03	0.008
Lake Mburo	−0.31	0.450	−3.37	0.064

## Discussion

### Sequence diversity and geographic population structure

Despite the wide distributional range, the fragmented nature of the habitat and low expected rate of long distance dispersal, our results show relatively little geographic differentiation among the southern populations of *B. anynana*. Towards the equator, the populations show more population structure, in particular the island population PE and the equatorial population LM. The molecular diversity indices are similar between the populations, including the island population Pemba for which lower diversity indices might have been expected due to its isolation from the mainland (Table 1). Haplotype diversity lies in the range 0.75 to 0.92, which is high when compared to many other species. Similarly high haplotype diversity values have been reported in other Lepidoptera species, such as the palearctic Small Tortoiseshell *Aglais urticae* [19] and the invasive horse-chestnut leaf miner *Cameraria ohridella* in Europe [20], but there are also reports of lower haplotype diversity, e.g. in the Monarch butterfly *Danaus plexippus* [21].

Although haplotype diversity is high, low nucleotide diversity values indicate only small differences between haplotypes. This is also evident from the minimum spanning haplotype network, which shows mostly single nucleotide differences between haplotypes (Figure 2). In terms of population differentiation, the haplotype network demonstrates that especially the populations FB, LP and ZO show relatively little divergence, and share the most common haplotype in the analysis. The southernmost populations, FB and LP, are especially closely related to each other as indicated by a very low and non-significant *F_ST_* value (Table 2). For these two populations, the majority of single haplotypes are only one or two nucleotides removed from the shared, most common, haplotype. The island population PE does not share the latter haplotype, but still shares three haplotypes with the other populations. The haplotypes for this population are closest related to those of WA, which is also geographically the nearest sampled population with a distance of approximately 170 km (the shortest distance to the coast from Pemba Island is 50 km). The LM population shows most differentiation, with only two haplotypes shared with one other population (PE). With a distance of over a 1000 km to the nearest sampled population, the LM population is geographically the most distant population in the analysis. Moreover, this population belongs to the subspecies *B. anynana centralis*, while the other populations are of the subspecies *B. anynana anynana*, a classification made on the basis of wing pattern morphology [6].

Sufficient gene flow between populations can slow down or prevent the process of geographic differentiation, and leave a signature of little population structure over large areas. This is commonly observed in flying insect species, specifically in those species that migrate or are good dispersers. Examples include Monarch butterflies *D. plexippus* [21], bumble bees *Bombus terrestris* [22], and dragonflies *Anax junius* [23]. In contrast, *B. anynana* are weak flyers and do not migrate, therefore it is unlikely that long distance dispersal occurs frequently in this species. Although differences between sequences are small, our data do indicate substantial geographic structure, in particular for the island population (PE) and the *B. anynana centralis* population (LM). This suggests that, at least for these two populations, gene flow is limited. Gene flow could play a role in the similarity between the mainland populations, and especially between the southernmost populations that show no significant geographic differentiation. However, it is most likely that a shared recent demographic history accounts for the major part of the observed phylogeographic pattern.

### Demographic history of the populations

The combination of high haplotype diversity and low nucleotide diversity, as observed in our data, can be a signature of a rapid demographic expansion from a small effective population size [2]. In recent years, statistical tests that were originally developed to test selective neutrality of mutations, have been implemented to detect such population growth [17]. These tests are generally based on the distribution of pair-wise differences between sequences within populations. Here, we chose to use two tests that are commonly used to detect population expansion and that differ somewhat in their approach. Tajima's *D* test [24] is based on the allele frequency distribution of segregating nucleotide sites. A positive value indicates a bias towards intermediate frequency alleles, while a negative value indicates a bias towards rare alleles, the latter being a signature of recent population expansion. Fu's *F_S_* test [18] is based on the distribution of alleles or haplotypes, and here too, negative values can indicate recent population growth. In the present study, Tajima's *D* test shows negative values for all populations, however, only the two southernmost populations FB and LP differ significantly from neutrality. Fu's *F_S_* test resulted in significant values for all populations except LM which was negative but not significant (Table 3). It has been shown that Fu's *F_S_* test is more powerful than Tajima's *D* [17], and this would explain the differences in significance for some populations. The overall negative values resulting from both tests indicate that there is an excess of rare mutations in the populations, which can imply recent population expansion. Alternatively, these values can result from balancing selection on a nearby locus, although studies demonstrating direct or indirect selection (through hitchhiking) on the mitochondrial genome in natural populations are rare, but see e.g. [25]. An analysis including additional neutral nuclear DNA markers could give a more complete perspective on the neutral population structure of the populations.

The explanation of recent demographic expansion corresponds well to the widely observed patterns of population expansion in organisms across taxa following the last glacial period, which ended around 12,500 years ago. Cooler and dryer conditions during the glacial maximum led to worldwide shifts and contraction of forest areas into small refugia, thereby reducing the area of available habitat for many species [26]. An extensive number of studies have provided genetic evidence of the glacial effects of glacial periods on population histories for various species in Europe and North America, but evidence for the African continent is less abundant [27]. Pollen data have revealed that tropical rain- and seasonal forests and dry woodland in Africa were reduced, and replaced by savanna vegetation during the last glacial maximum [26], [28]. It has also been shown that several taxa of tropical rain forest vegetation persisted in equatorial Africa during this period [29], indicating the existence of forest refugia. Studies on butterflies, birds, reptiles, mammals and other animals support this theory [30], [31]. Based on our results, which indicate increasingly recent population expansion towards the South, it is likely that the distributional range of *B. anynana* species expanded southwards during the Holocene from glacial equatorial habitat refugia.

In conclusion, our study reveals a general high genetic diversity within populations of *B. anynana*, but relatively little differentiation among the southern populations, especially when taking into account the limited dispersal ability of the species and the fragmented nature of the habitat. The observed patterns of genetic variation within and between the populations are most likely caused by a recent shared demographic history in the form of a reduced species area in the last glacial period. Interestingly, despite the indication that the populations underwent recent expansion from ice age refugia, the species shows population differentiation in wing pattern, not only for the relatively isolated populations of the subspecies *B. anynana centralis* [6] and Pemba Island (M.A. de Jong, personal observation), but also for more closely related populations on the mainland [4]. These findings suggest that despite recent population history, population differentiation in morphology and potentially in other traits may occur relatively rapidly in *B. anynana*. This study provides a much needed framework for ongoing investigation of adaptive functional variation at the phenotypic or molecular level in wild populations of *B. anynana*.

## References

[1] Slatkin M (1987). Gene flow and the geographic structure of natural populations.. Science.

[2] Avise JC (2000). Phylogeography: the history and formation of species.

[3] Oostra V, de Jong MA, Invergo B, Kesbeke FMNH, Wende F (2010). Translating environmental gradients into discontinuous reaction norms via hormone signalling in a polyphenic butterfly.. Proc R Soc B.

[4] de Jong MA, Kesbeke FMNH, Brakefield PM, Zwaan BJ (2010). Geographic variation in thermal plasticity of life history and wing pattern in *Bicyclus anynana*.. Clim Res.

[5] Brakefield PM (2010). Radiations of Mycalesine butterflies and opening up their exploration of morphospace.. Am Nat.

[6] Condamin M (1973). Monographie du genre *Bicyclus* (Lepidoptera Satyridae).. Mémoires de l'Institute Fondamental d'Afrique Noire.

[7] Brakefield PM, Reitsma N (1991). Phenotypic plasticity, seasonal climate and the population biology of *Bicyclus* butterflies (Satyridae) in Malawi.. Ecol Entomol.

[8] Brakefield PM, Gates J, Keys D, Kesbeke F, Wijngaarden PJ (1996). Development, plasticity and evolution of butterfly eyespot patterns.. Nature.

[9] Moritz C, Dowling TE, Brown WM (1987). Evolution of animal mitochondrial DNA: relevance for population biology and systematics.. Annu Rev Ecol Syst.

[10] Harrison RG (1989). Animal mitochondrial DNA as a genetic marker in population and evolutionary biology.. Trends Ecol Evol.

[11] Caterino MS, Soowon C, Sperling FAH (2000). The current state of insect molecular systematics: a thriving tower of Babel.. Annu Rev Entomol.

[12] Wahlberg N, Wheat CW (2008). Genomic outposts serve the phylogenomic pioneers: designing novel nuclear markers for genomic DNA extractions of Lepidoptera.. Syst Biol.

[13] Hall TA (1999). BioEdit: a user-friendly biological sequence alignment editor and analysis program for Windows 95/98/NT.. Nucl Acid S.

[14] Excoffier L, Lischer HEL (2010). Arlequin suite ver 3.5: A new series of programs to perform population genetics analyses under Linux and Windows.. Mol Ecol Res.

[15] Nei M (1987). Molecular evolutionary genetics.

[16] Excoffier L, Smouse PE, Quattro JM (1992). Analysis of molecular variance inferred from metric distances among DNA haplotypes: application to human mitochondrial DNA restriction data.. Genetics.

[17] Ramos-Onsins SE, Rozas J (2002). Statistical properties of new neutrality tests against population growth.. Mol Biol Evol.

[18] Fu Y-X (1997). Statistical tests of neutrality of mutations against population growth, hitchhiking and background selection.. Genetics.

[19] Vandewoestijne S, Baguette M, Brakefield PM, Saccheri IJ (2004). Phylogeography of *Aglais urticae* (Lepidoptera) based on DNA sequences of the mitochondrial COI gene and control region.. Mol Phylogenet Evol.

[20] Valade R, Kenis M, Hernandez-Lopez A, Augustin S, Mari Mena N (2009). Mitochondrial and microsatellite DNA markers reveal a Balkan origin for the highly invasive horse-chestnut leaf miner *Cameraria ohridella* (Lepidoptera, Gracillariidae).. Mol Ecol.

[21] Brower AVZ, Boyce TM (1991). Mitochondrial DNA variation in monarch butterflies.. Evolution.

[22] Estoup A, Solignac M, Cornuet JM, Goudet J, Scholl A (1996). Genetic differentiation of continental and island populations of *Bombus terrestris* (Hymenoptera: Apidae) in Europe.. Mol Ecol.

[23] Freeland JR, May M, Lodge R, Conrad KF (2003). Genetic diversity and widespread haplotypes in a migratory dragonfly, the common green darner *Anax junius*.. Ecol Entomol.

[24] Tajima F (1989). Statistical method for testing the neutral mutation hypothesis by DNA polymorphism.. Genetics.

[25] Ruiz-Pesini E, Mishmar D, Brandon M, Procaccio V, Wallace DC (2004). Effects of purifying and adaptive selection on regional variation in human mtDNA.. Science.

[26] Prentice IC, Jolly D (2000). Mid-Holocene and glacial-maximum vegetation geography of the northern continents and Africa.. J Biogeogr.

[27] Hewitt G (2000). The genetic legacy of the Quaternary ice ages.. Nature.

[28] Flenley JR (1998). Tropical forests under the climates of the last 30,000 years.. Climate Change.

[29] Elenga H, Peyron O, Bonnefille R, Prentice IC, Jolly D (2000). Pollen-based biome reconstructions for southern Europe and Africa 18,000 yr bp.. J Biogeogr.

[30] Hamilton A, Taylor D, Howard P, Weber W, White LJT, Vedder A, Naughton-Treves L (2001). Hotspots in African forests as Quaternary refugia.. African rain forest ecology and conservation: an interdisciplinary perspective.

[31] Douglass JF, Miller LD (2003). Afrotropical skippers (Lepidoptera: Hesperioidea) and the emergence of the combined refugium theory.. Bulletin of the Allyn Museum.

